# Isolation, Characterization and Biological Properties of Membrane Vesicles Produced by the Swine Pathogen *Streptococcus suis*

**DOI:** 10.1371/journal.pone.0130528

**Published:** 2015-06-25

**Authors:** Bruno Haas, Daniel Grenier

**Affiliations:** 1 Groupe de Recherche en Écologie Buccale (GREB), Faculté de Médecine Dentaire, Université Laval, Quebec City, QC, Canada; 2 Centre de Recherche en Infectiologie Porcine et Avicole (CRIPA), Fonds de Recherche du Québec—Nature et Technologies (FRQNT), Saint-Hyacinthe, QC, Canada; University of South Dakota, UNITED STATES

## Abstract

*Streptococcus suis*, more particularly serotype 2, is a major swine pathogen and an emerging zoonotic agent worldwide that mainly causes meningitis, septicemia, endocarditis, and pneumonia. Although several potential virulence factors produced by *S*. *suis* have been identified in the last decade, the pathogenesis of *S*. *suis* infections is still not fully understood. In the present study, we showed that *S*. *suis* produces membrane vesicles (MVs) that range in diameter from 13 to 130 nm and that appear to be coated by capsular material. A proteomic analysis of the MVs revealed that they contain 46 proteins, 9 of which are considered as proven or suspected virulence factors. Biological assays confirmed that *S*. *suis* MVs possess active subtilisin-like protease (SspA) and DNase (SsnA). *S*. *suis* MVs degraded neutrophil extracellular traps, a property that may contribute to the ability of the bacterium to escape the host defense response. MVs also activated the nuclear factor-kappa B (NF-κB) signaling pathway in both monocytes and macrophages, inducing the secretion of pro-inflammatory cytokines, which may in turn contribute to increase the permeability of the blood brain barrier. The present study brought evidence that *S*. *suis* MVs may play a role as a virulence factor in the pathogenesis of *S*. *suis* infections, and given their composition be an excellent candidate for vaccine development.

## Introduction

Bacterial membrane vesicles were first discovered some five decades ago in Gram-negative bacteria [1]. These globular structures, which range in diameter from 10 to 200 nm, result from outer membrane blebbing and are called outer membrane vesicles (OMV). The biogenesis of OMVs remains unclear due to the difficulty in obtaining mutant strains that do not produce OMVs [2]. Over the years, many roles have been assigned to OMVs, including intercellular communication [3], response to environmental stresses [4], biofilm formation [5], pathogenic processes [6], and horizontal gene transfer [7]. The rising interest in bacterial membrane vesicles lead to the creation of EVpedia, a web-based database that collects published data about vesicles produced by prokaryotes and eukaryotes [8]. Given that OMVs contain large amounts of membrane-associated proteins and virulence factors [9], they are highly immunogenic and may induce immunoprotection against pathogens. This has led to a growing interest in bacterial OMVs as potential vaccine candidates [10]. An OMV-based *Neisseria meningitidis* vaccine has already proven its effectiveness and is currently being used to prevent meningitis [11].

Despite the fact that vesicles produced by Gram-negative bacteria have been extensively studied, vesicles from Gram-positive bacteria were overlooked for decades since it was thought that the rigidity of the peptidoglycan-rich cell wall would not allow vesicle blebbing [12]. The discovery of cytoplasmic membrane-derived vesicles, or simply membrane vesicles (MVs), in Gram-positive bacteria, suggested that vesicle production is a ubiquitous phenomenon [13]. They were first described in 1990 [14] and studied in more depth in the last decade. MV production by numerous Gram-positive bacteria as well as their putative roles in virulence have been reported for *Staphylococcus aureus* [12], *Bacillus* spp. [15, 16], *Listeria monocytogenes* [17], *Clostridium perfringens* [18], *Streptomyces coelicolor* [19] and *Streptococcus* spp. [20, 21] (for recent review, see [22]).

The Gram-positive bacterium *Streptococcus suis* is a major swine pathogen worldwide that mainly causes septicemia, meningitis, endocarditis, arthritis, and pneumonia [23]. It is also considered an emerging zoonosis agent since it can infect humans who are in close contact with diseased pigs or their byproducts [24]. Thirty-three serotypes have been described to date based on the composition of their capsular polysaccharides. While the serotype distribution varies depending on the geographical origins of the strains, *S*. *suis* serotype 2 is considered the most pathogenic and the most prevalent capsular type recovered from diseased pigs and humans [23, 24]. The massive use of antibiotics in the swine industry has contributed to the emergence of drug resistant strains, making *S*. *suis* infections more difficult to treat and causing increasingly severe economic losses [25]. It is thus essential to better understand the pathogenic process of *S*. *suis* infections in order to identify new therapeutic strategies.

In this study, we hypothesized that *S*. *suis* produces MVs that may play an important role in the pathogenesis of *S*. *suis* infections. MVs were isolated using a differential centrifugation and filtration protocol. Their protein composition was analyzed and characterized using a proteomic approach. Given that bacterial vesicles generally carry virulence factors, two virulence-associated activities were assayed. The pro-inflammatory effects of *S*. *suis* MVs on monocytes and macrophages were also investigated.

## Materials and Methods

### Bacterial strain and isolation of membrane vesicles

*S*. *suis* P1/7 was grown in Todd-Hewitt Broth (THB, Becton, Dickinson and Company, Sparks, MD, USA) at 37°C. MVs were isolated using centrifugation and filtration protocol as described by Lee *et al*. [12], with slight modifications. Briefly, a 1-L overnight culture of *S*. *suis* (early stationary phase) was centrifuged at 10 000 × g for 20 min at 4°C, and the supernatant was filtered through a 0.45-μm pore size cellulose nitrate membrane and then through a 0.22 μm pore size cellulose nitrate membrane to remove residual bacteria. The filtrate was centrifuged at 150 000 × g for 3 h at 4°C. The pellets were resuspended in 50 mM phosphate-buffered saline (PBS, pH 7.2), further centrifuged similarly, and stored in PBS at –20°C until used. MVs were quantified by dry weight as well as by protein content (Quick Start Bradford Assay; Bio-Rad Laboratories, Mississauga, ON, Canada) according to the manufacturer’s protocol.

### Transmission electron microscopy

Vesicles were suspended in 0.1 M sodium cacodylate buffer (pH 7) containing 5% glutaraldehyde and 0.15% ruthenium red. They were then treated with 1 mg/ml of polycationic ferritin and were processed as previously described by Vanrobaeys *et al*. [26]. Thin sections were prepared and were observed using a JEOL 1230 transmission electron microscope at an accelerating voltage of 80 kV. A 1.27 correction factor was applied to determine MVs diameter, as suggested by Kong *et al*. [27].

### Proteomic analysis

Three independent batches of *S*. *suis* MVs with a protein content of 10 μg were washed three times with 50 mM ammonium bicarbonate buffer (pH 8) using Amicon 3-kDa molecular weight cut off centrifugal filters (EMD Millipore Canada; Mississauga, ON, Canada). The samples were vacuum-dried in a Speedvac concentrator (Thermo Fisher Scientific, Waltham, MA, USA) and were stored at –20°C until they were trypsin digested. Proteins were solubilized in 25 μl of 50 mM ammonium bicarbonate containing 1% sodium deoxycholate and were heated at 95°C for 5 min. The samples were reduced by adding 0.2 mM dithiothreitol (37°C for 30 min) and were alkylated by adding 0.9 mM iodoacetamide (37°C for 20 min). Trypsin (1 μg) was then added, and the samples were incubated overnight at 37°C. Proteolytic degradation was stopped by acidification with 3% acetonitrile-1% trifluoroacetic acid (TFA)-0.5% acetic acid. Peptides were purified using C18 spin tips (Thermo Fisher Scientific) and were pooled, vacuum centrifuge-dried, and resuspended in 0.1% formic acid. Peptide samples (800 ng) were fractionated by online reversed-phase nanoscale capillary liquid chromatography (nanoLC). The fractions were analyzed by electrospray mass spectrometry (ES MS/MS; Proteomics platform of the Québec Genomic Center) using a 5600 mass spectrometer (AB Sciex, Framingham, MA, USA) equipped with an Agilent 1200 nano pump, a nanoelectrospray ion source, and a self-pack PicoFrit column (15 cm x 0.075 i.d.; New Objective, Woburn, MA, USA) packed with a Jupiter C18 phase (particle size: 5 μm; pore size: 300 Å; Phenomenex, Torrance, CA). Peptides were eluted with a linear 2–50% linear gradient of acetonitrile-0.1% formic acid for 30 min at 300 nl/min. Mass spectra were acquired using Analyst software version 1.6 with the data dependent acquisition mode (AB Sciex). Each full scan mass spectrum (400 to 1250 m/z) was followed by collision-induced dissociation of the twenty most intense ions. The dynamic exclusion was set at 3 s and the tolerance at 100 ppm.

MS/MS peak list Mascot Generic Format (MGF) files were generated using ProteinPilot version 4.5 (AB Sciex) with the Paragon and Progroup algorithms [28]. The MGF sample files were analyzed using Mascot version 2.4.0 (Matrix Science, London, UK). Mascot and X! Tandem were set up to search the UniRef StreptococcusSuisP17 database (version February 2014, 2039 entries) assuming trypsin digestion. Mascot and X! Tandem were searched with a fragment ion mass tolerance of 0.100 Da and a parent ion tolerance of 0.100 Da. Carbamidomethylated cysteine was specified in Mascot and X! Tandem as a fixed modification. Dehydrated N-terminus, Glu->pyro-Glu of the N-terminus, ammonia-loss of the N-terminus, Gln->pyro-Glu of the N-terminus, deamidated asparagine and glutamine, and oxidation of methionine were specified in X! Tandem as variable modifications.

Proteome Scaffold version 4.3.4 (Proteome Software Inc., Portland, OR USA) was used to validate the MS/MS-based peptide and protein identification. Peptide identification was accepted if it could established there was a 6% or greater probability that the False Discovery Rate (FDR) was less than 1% using the Peptide Prophet algorithm [29] with Scaffold delta-mass correction. Peptide identification was accepted if it could be established that there was a 98% or greater probability of achieving an FDR less than 1% and that the protein contained at least three identified peptides. Proteins were considered present in significant amounts when at least 30 spectra were identified. The relative abundance of proteins was determined by spectral counting. Protein probabilities were assigned by the Protein Prophet algorithm [30]. Proteins that contained similar peptides and could not be differentiated based on an MS/MS analysis alone were grouped to satisfy the principles of parsimony. Proteins were annotated with Gene Ontology (GO) terms from gene_association.goa_uniprot (downloaded March 18, 2013) [31]. Protein localization was predicted using GO terms as well as the Uniprot database. Proteins containing a LPXTG motif were predicted as cell wall-anchored.

### Determination of enzymatic activities

N-succinyl-Ala-Ala-Pro-Phe-pNa, the substrate for the *S*. *suis* subtilisin protease SspA, was dissolved at a concentration of 2 mg/ml in 50% dimethylformamide, and 20 μl was added to the wells of a 96-well microplate containing 100 μl of *S*. *suis* MVs (protein content of 300 and 80 μg/ml in PBS). Assay mixtures were incubated at 37°C for 4 h. The absorbance at 415 nm (A_415_) was then recorded using a xMark microplate reader (Bio-Rad Laboratories). DNase activity was assayed as described in a previous study [32]. Briefly, MVs (protein content of 64, 32, and 16 μg/ml in PBS, pH 7.5) were incubated for 4 h at 37°C in the presence of 2 μg of salmon sperm DNA (Sigma-Aldrich Canada Ltd., Oakville, ON, Canada) in 50 mM PBS. One hundred microliters of Quant-iT Pico-Green dsDNA reagent (Life Technologies Inc., Burlington, ON, Canada) prepared following the manufacturer’s protocol was added to the reaction mixtures, which were then incubated for 5 min at room temperature. Fluorescence was recorded using a Synergy 2 microplate reader (BioTek Instruments, Winooski, VT, USA) with the excitation and emission wavelengths set at 485 ± 20 nm and 528 ± 20 nm, respectively.

### Neutrophil extracellular traps degradation

The human promyelocytic leukemia cell line HL60 (ATCC CCL-240) was routinely grown in RPMI-1640 medium (Life Technologies Inc.) supplemented with 10% heat-inactivatedfetal bovine serum (FBS) and 100 μg/ml of penicillin-streptomycin at 37°C in a 5% CO_2_ atmosphere. Cells (10^6^ cells/ml) were treated for 3 days with 1.25% dimethylsulfoxide (DMSO) to induce differentiation into neutrophil-like cells. The efficacy of cell differentiation was evaluated using the nitro-blue tetrazolium (NBT) assay according to Collins *et al*. [33], with modifications. Briefly, 500 μl of cells (5.10^6^ cells/ml) were incubated for 2 h at 37°C in the presence of 10 μg/ml of *Escherichia coli* lipopolysaccharide (LPS) and 250 μl of 0.01% NBT (Sigma-Aldrich Canada Ltd.). The reaction was stopped by adding 500 μl of 0.5 M HCl. The mixtures were centrifuged for 4 min at 10 000 × g, and the pellets were resuspended in 200 μl of DMSO. The absorbance at 550 nm (A_550_) was recorded using a xMark microplate reader (Bio-Rad Laboratories) and was compared to a negative control (non-differentiated cells). Neutrophil-like cells (100 μl, 10^6^ cells/ml) were seeded in a 96-well black wall clear bottom microplate and were treated with 0.5 μM phorbol 12-myristate 13-acetate (PMA) for 3 h at 37°C in a 5% CO_2_ atmosphere. MVs (protein content of 40, 20, 10, or 1 μg/ml) were then added to the reaction mixtures, and the samples were incubated for an additional 2 h. Extracellular DNA was labeled using 0.5 μM SYTOX Green Nucleic Acid Stain (Life Technologies Inc.) according to the manufacturer’s instructions. After a 10-min incubation at room temperature, fluorescence was recorded using a Synergy 2 microplate reader (BioTek Instruments) with the excitation and emission wavelengths set at 485 ± 20 nm and 528 ± 20 nm, respectively. Unstimulated cells were used as blanks to subtract the background signal.

### Activation of the NF-κB signaling pathway in monocytes and macrophages

The human monoblastic leukemia cell line U937-3xκB-LUC (U937 cells stably transfected with a construct containing 3 NF-κB binding sites from the Ig κ light chain promoter coupled with the gene encoding the firefly luciferase (3x-κB-*luc*) [34]) was kindly provided by Dr. Rune Blomhoff (University of Oslo, Norway). The cells were routinely grown at 37°C in a 5% CO_2_ atmosphere in RPMI-1640 medium supplemented with 10% FBS, 100 μg/ml of penicillin-streptomycin, and 75 μg/ml of hygromycin B (Sigma-Aldrich Canada Ltd.). The monocytes (2.10^5^ cells/ml) were differentiated into macrophage-like cells by incubating them with 10 ng/ml PMA for 24 h. The cell culture medium was then replaced with fresh medium, and the adherent macrophage-like cells were incubated for an additional 24 h prior to use. U937-3xκB-LUC monocytes and macrophage-like cells were suspended at a concentration of 2.10^6^ cells/ml in RPMI-1640 supplemented with 1% FBS, 100 μg/ml of penicillin-streptomycin, and 75 μg/ml of hygromycin B. The cell suspensions (50 μl) were seeded into wells of black bottom, black wall 96-well microplates. Two-fold serial dilutions of *S*. *suis* MVs (protein content ranging from 80 to 0.02 μg/ml) were prepared in cell culture medium, and 50 μl of each dilution was added to the wells. The microplates were incubated for 6 h at 37°C in a 5% CO_2_ atmosphere. *E*. *coli* LPS (10 μg/ml) and *S*. *suis* P1/7 whole cells (multiplicity of infection [MOI] of 100) were used as positive controls. NF-κB activation was monitored using the Bright-Glo Luciferase Assay System (Promega, Madison, WI, USA) by adding 100 μl of luciferase substrate to the wells at room temperature. Luminescence was recorded using the luminometer option of a Synergy 2 microplate reader (BioTek Instruments) within 3 min of the addition of the substrate.

### Cytokine secretion by macrophages

The human monoblastic leukemia U937 cell line was cultivated in RPMI-1640 supplemented with 10% FBS and 100 μg/ml of penicillin-streptomycin at 37°C in a 5% CO_2_ atmosphere. The cells were then differentiated into adherent macrophage-like cells as described above. Macrophage-like cells (10^6^ cells/ml) were seeded in 6-well cell culture plates (2 ml/well) and were incubated overnight to allow adherence. The cell culture medium was aspirated and was replaced with *S*. *suis* P1/7 cell suspensions at an MOI of 100 (positive control) or *S*. *suis* MVs (protein content of 40, 20, 10, or 1 μg/ml) prepared in RPMI-1640 supplemented with 1% FBS and 100 μg/ml of penicillin-streptomycin. Following a 24-h incubation at 37°C in a 5% CO_2_ atmosphere, the culture supernatants were collected and were used to quantify interleukin (CXCL)-8, tumor necrosis factor (TNF)-α, and interleukin (IL)-1β using enzyme-linked immunosorbent assay (ELISA) kits following the manufacturer’s protocols (eBioscience Inc., San Diego, CA, USA).

### Statistical analysis

All assays were performed in triplicate, and the means ± standard deviations were calculated. Differences were analyzed for statistical significance using the Student’s t-test and were considered significant at *p* < 0.05.

## Results

**Fig 1:**
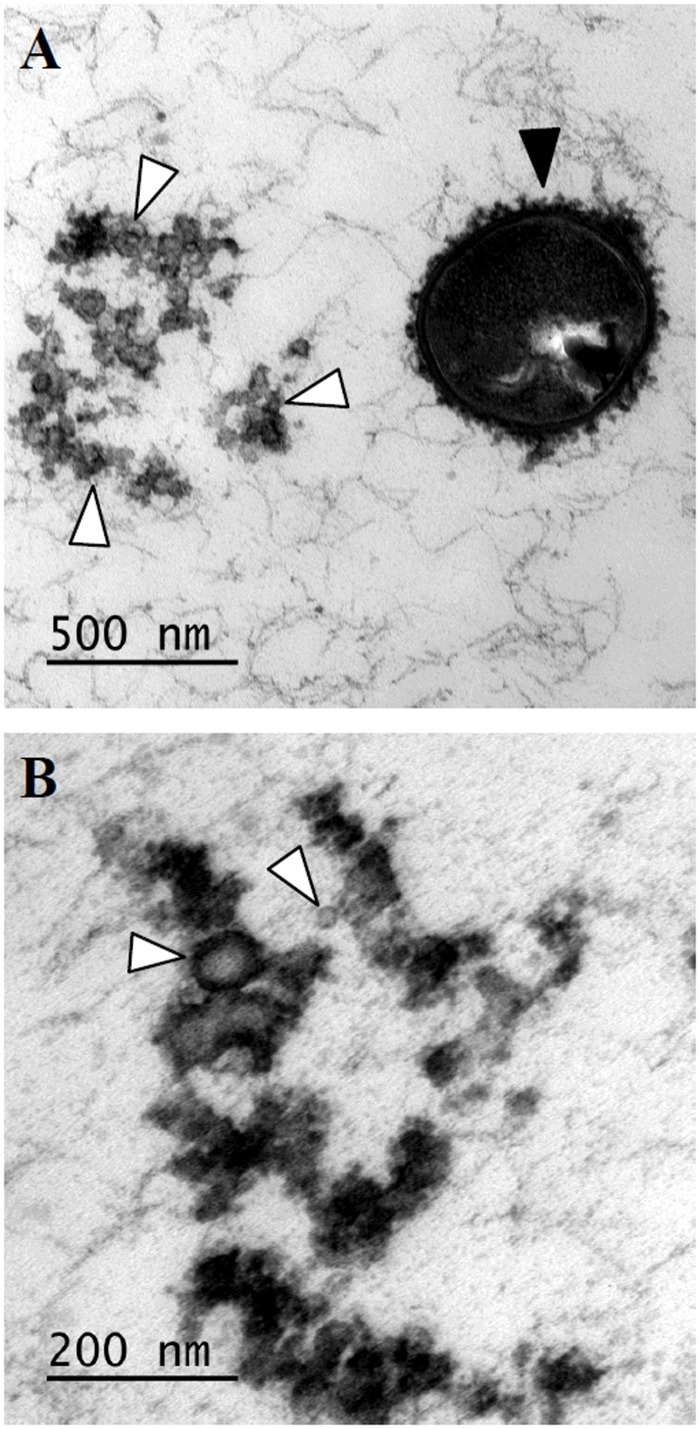
Transmission electron micrographs of an overnight culture of *S*. *suis* P1/7 (panel A) and the membrane vesicle preparation (panel B). Black arrow: whole bacterium; white arrows: membrane vesicles.

Transmission electron microscopic observations of overnight cultures of *S*. *suis* P1/7 revealed the presence of membranous globular structures, suggesting that *S*. *suis* P1/7 produces MVs (Fig 1A). The MVs were isolated using a differential centrifugation and filtration protocol. The average recovery rate was 4 mg of MVs (dry weight) per liter of overnight culture, which corresponded to approximately 300 μg of vesicular protein content. As shown in Fig 1B, the MVs appeared to be coated with a dense structure, most likely capsular material, and ranged from 13 to 130 nm in diameter.

**Fig 2:**
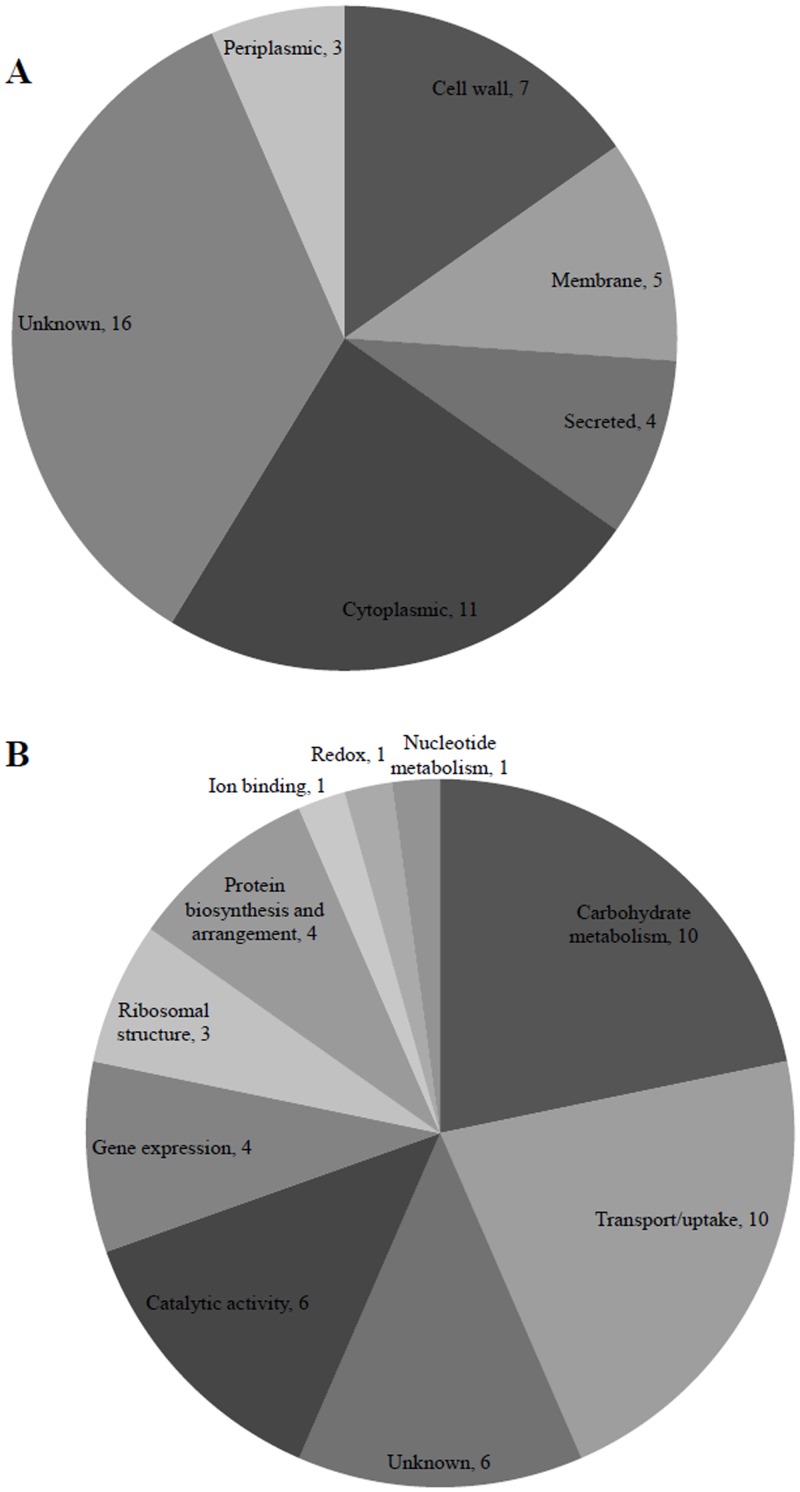
General locations (A) and functions (B) of proteins identified in *S. suis* P1/7 membrane vesicles by ES MS/MS.

Proteomic sequencing detected 46 proteins in *S*. *suis* P1/7 MVs, which are listed in Table 1 by their abundance in each functional group. Only proteins that were detected in at least two batches of MVs and counting 30 or more spectra were considered. Proteins identified using the *S*. *suis* P1/7 genome database were either cytoplasmic (n = 11), membrane/cell wall-associated (n = 12), periplasmic (n = 3) or secreted (n = 4). However, the location of 16 proteins could not be assigned (Fig 2A). The biological activities associated with *S*. *suis* P1/7 vesicular proteins were mostly involved in the carbohydrate metabolism (n = 10), molecule transport (n = 10), catalytic activities (n = 6), gene expression (n = 4), ribosomal structure (n = 3), protein biosynthesis and arrangement (n = 4), ion binding (n = 1), oxydoreduction (n = 1), nucleotide metabolism (n = 1), and 6 proteins were of unknown function (Fig 2B). In addition, 9 proteins (19.6%) were identified as proven or suspected virulence factors (Table 2), seven of which were located at the cell surface (either cell wall- or membrane-associated) and two were secreted. For the 46 proteins, in terms of relative abundance, the subtilisin-like protease SspA predominated in the MVs, followed by a putative lipoprotein (component of an ABC transporter), the cell wall-anchored DNase SsnA, the IgM protease Ide, putative exported ribonucleases (G and E), a putative N-acetylmuramoyl-L-alanine amidase, the Plr adhesin (glyceraldehyde-3-phosphate dehydrogenase), a putative sugar binding protein, the peptide binding protein OppA, the histidine triad-family protein HtpsC, the muraminidase-released protein Mrp, the elongation factor Tu, a fumarate reductase flavoprotein subunit, an unidentified putative exported protein, and a putative ABC transporter component. Interestingly, of the 15 most abundant proteins identified in the MVs, six are considered virulence factors (SspA, SsnA, Ide, Plr, HtpsC, and Mrp).

**Table 1:** Protein content of *S*. *suis* P1/7 membrane vesicles identified by ES MS/MS in order of abundance in each functional group.

Protein accession number	Gene accession number	MS/MS identification	Molecular weight	Protein name/function	Predicted localization
**Translational**, **ribosomal structure**, **and biogenesis (n = 3)**					
C5VVU1	SSU0721	Putative 30S ribosomal protein S1	44 kDa	30S Ribosomal protein S1	Cytoplasm
C5VWE9	SSU1935	30S ribosomal protein S4	23 kDa	30S Ribosomal protein S4	Cytoplasm
C5VVK7	SSU1771	30S ribosomal protein S2	29 kDa	30S Ribosomal protein S2	Cytoplasm
**Transport**, **uptake**, **and osmoregulation (n = 9)**					
C5VWT7	SSU0934	Putative lipoprotein	36 kDa	Putative ABC transporter	Membrane
C5W096	SSU1664	Putative oligopeptide-binding protein OppA	66 kDa	OppA / Peptide binding	Unknown
C5W029	SSU0503	Putative amino acid ABC transporter	31 kDa	ABC transporter	Unknown
C5VW03	SSU1853	Putative amino-acid ABC transporter	29 kDa	ABC transporter	Periplasm
C5VXJ7	SSU1170	Extracellular solute-binding protein	61 kDa	Sugar ABC transporter	Secreted
C5VW19	SSU1869	Extracellular metal cation-binding protein	36 kDa	ZnuA / Zinc uptake	Secreted
C5VZZ1	SSU0465	Putative exported protein	41 kDa	Predicted membrane fusion protein	Membrane
C5VYV7	SSU0284	Extracellular solute-binding protein	35 kDa	ABC transporter	Periplasm
C5VYN9	SSU1364	Branched-chain amino acid ABC transporter	41 kDa	ABC transporter	Periplasm
**Protein biosynthesis (n = 2)**					
C5VXU6	SSU1196	Threonine-tRNA ligase	75 kDa	ThrS / protein biosythesis	Cytoplasm
C5VZL9	SSU0412	Valine-tRNA ligase	101 kDa	ValS / protein biosynthesis	Cytoplasm
**Carbohydrate/sugar metabolism (n = 10)**					
C5VXF6	SSU1127	Putative N-acetylmuramoyl-L-alanine amidase	113 kDa	Peptidoglycan catabolism	Unknown
C5VY36	SSU0153	Glyceraldehyde-3-phosphate dehydrogenase	39 kDa	Plr / adhesin	Secreted
C5VWC9	SSU1915	Putative maltose/maltodextrin-binding protein	44 kDa	sugar binding	Membrane
C5VWE7	SSU1933	Putative fumarate reductase flavoprotein subunit	53 kDa	Fumarate reductase flavoprotein subunit	Unknown
C5VXV7	SSU1207	Putative lipoprotein	30 kDa	Predicted glucose-6-phosphate isomerase	Unknown
C5VVZ9	SSU1849	Putative surface-anchored amylopullulanase	234 kDa	ApuA / amylopullulanase / Adhesin	Cell wall
C5VY37	SSU0154	Phosphoglycerate kinase	42 kDa	Phosphoglycerate kinase	Cytoplasm
C5VYJ5	SSU1320	Enolase	47 kDa	Eno / adhesin	Cell wall
C5VYP7	SSU1372	Multiple sugar-binding protein	45 kDa	Sugar transporter	Unknown
C5VZ34	SSU1451	2,3-bisphosphoglycerate-dependent phosphoglycerate mutase	26 kDa	GpmA / glycolytic process	Unknown
**Nucleotide metabolism (n = 1)**					
C5VX44	SSU1044	Ribonucleoside-diphosphate reductase	82 kDa	DNA synthesis	Cytoplasm
**Protein folding/arranging (n = 2)**					
C5VX75	SSU1078	Foldase protein PrsA	38 kDa	PrsA / protein folding	Membrane
C5VXK4	SSU1177	Peptidyl-prolyl cis-trans isomerase	28 kDa	Protein folding	Unknown
**Gene expression (n = 4)**					
C5W008	SSU0482	Elongation factor Tu	44 kDa	TufA / elongation factor Tu	Cytoplasm
C5VY08	SSU0123	DNA-directed RNA polymerase subunit beta	136 kDa	DNA-dependant RNA polymerase	Cytoplasm
C5VY34	SSU0151	Elongation factor G	77 kDa	FusA / elongation factor G	Cytoplasm
C5VY07	SSU0122	DNA-directed RNA polymerase subunit beta	133 kDa	RpoB / RNA-polymerase	Cytoplasm
**Catalytic activity (n = 5)**					
C5VVK9	SSU1773	Putative surface-anchored serine protease	187 kDa	SspA / Subtilisin-like protease	Cell wall
C5VVJ6	SSU1760	Surface-anchored DNA nuclease	114 kDa	SsnA / Nuclease	Cell wall
C5W022	SSU0496	Putative Mac family protein	124 kDa	IgM protease	Membrane
C5W049	SSU1616	Putative exported protein	122 kDa	Predicted ribonuclease G and E	Unknown
C5VXH2	SSU1143	Putative surface-anchored zinc carboxypeptidase	118 kDa	Surface anchored zinc carboxypeptidase	Cell wall
**Defense/virulence mechanisms (n = 4)**					
C5VYR5	SSU1390	Streptococcal histidine triad-family protein	96 kDa	HtpsC	Cell wall
C5VVS8	SSU0706	Muramidase-released protein	136 kDa	Mrp	Cell wall
C5VZA8	SSU0370	Putative penicillin-binding protein 1A	79 kDa	Pbp1A / Penicillin-binding protein	Unknown
C5VXY1	SSU1231	Suilysin	55 kDa	Sly / Hemolysin	Secreted
**Ion binding (n = 1)**					
C5VY00	SSU0115	Zinc-binding protein AdcA	56 kDa	AdcA/Zinc-binding protein	Unknown
**Redox (n = 1)**					
C5VYT4	SSU0261	Aldehyde-alcohol dehydrogenase	97 kDa	Oxydoreductase	Unknown
**Uncharacterized proteins (n = 4)**					
C5VV38	SSU0595	Putative exported protein	13 kDa		Unknown
C5VZS6	SSU1560	Putative lipoprotein	17 kDa		Unknown
C5VV37	SSU0594	Putative exported protein	14 kDa		Unknown
C5VV35	SSU0592	Putative exported protein	12 kDa		Unknown

**Table 2:** Virulence factors identified in *S*. *suis* P1/7 membrane vesicles.

Identified proteins	Factor	Protein accession number	Gene accession number	Molecular weight	Function	References
Surface-anchored serine protease	SspA	C5VVK9	SSU1773	187 kDa	Subtilisin-like protease	[39, 40]
Surface-anchored DNA nuclease	SsnA	C5VVJ6	SSU1760	114 kDa	DNase	[41, 42]
Mac family protein	Ide	C5W022	SSU0496	124 kDa	IgM protease	[38]
Glyceraldehyde-3-phosphate dehydrogenase	Plr	C5VY36	SSU0153	39 kDa	Adhesin	[46]
Streptococcal histidine triad-family protein	HtpsC	C5VYR5	SSU1390	96 kDa	Unknown	[37]
Muramidase-released protein	Mrp	C5VVS8	SSU0706	136 kDa	Unknown	[43]
Surface-anchored amylopullulanase	ApuA	C5VVZ9	SSU1849	234 kDa	Adhesin	[44]
Enolase	Eno	C5VYJ5	SSU1320	47 kDa	Adhesin	[45]
Suilysin	Sly	C5VXY1	SSU1231	55 kDa	Hemolysin	[47, 48]

**Fig 3:**
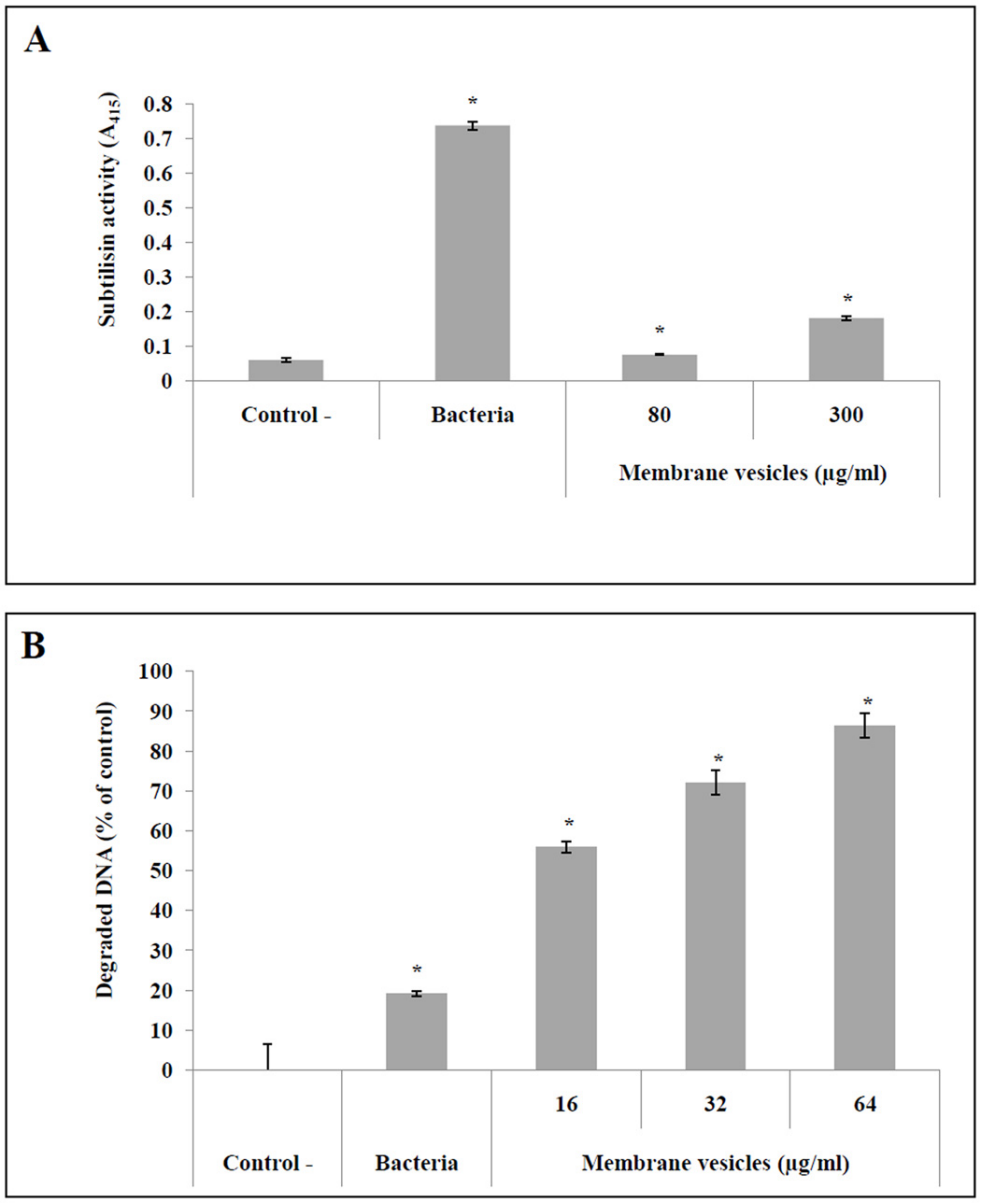
Determination of subtilisin (A) and DNase (B) activities in *S. suis* membrane vesicles. *: *p* < 0.05 compared to negative controls.

Based on the subtilisin and DNase assays performed to confirm the presence of active virulence factors in *S*. *suis* P1/7 MVs, only the highest concentration of MVs (300 μg/ml in protein content) displayed subtilisin activity (Fig 3A). On the other hand, the MVs dose-dependently degraded DNA (Fig 3B). More specifically, at a concentration of 64 μg/ml in protein content, the MVs degraded approximately 90% of the DNA whereas whole bacteria (OD_660_ = 1) only degraded 20% after a 4 h incubation.

**Fig 4:**
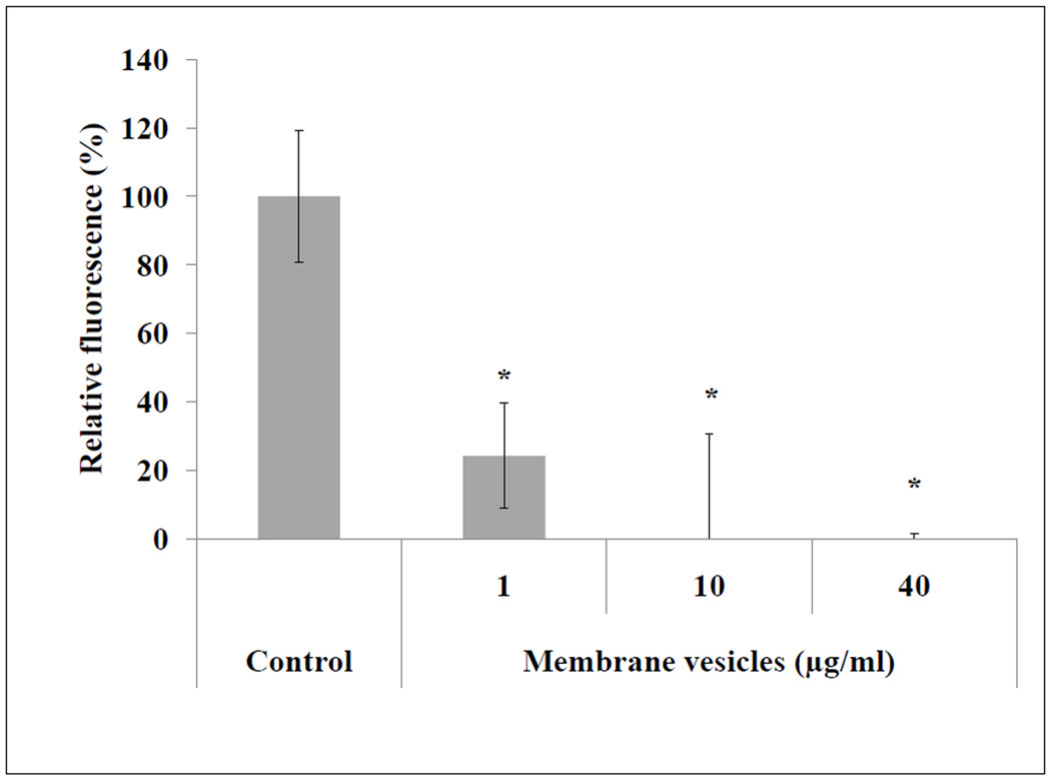
Quantification of NET degradation by *S*. *suis* membrane vesicles. NETs were formed by the PMA-stimulated promyelocytic leukemia cell line HL60. *: *p* < 0.05 compared to the negative control (no NET degradation).

Given that *S*. *suis* MVs possessed strong DNase activity, we hypothesized that such structures may induce the degradation of neutrophil extracellular traps (NETs), which consist of DNA as a backbone with embedded histones, antimicrobial peptides, and proteases, and which are part of the first innate immune response at sites of infection [35]. As shown in Fig 4, MVs at a concentration as low as 1 μg/ml in protein content caused the degradation of approximately 70% of extracellular DNA excreted by NETs.

**Fig 5:**
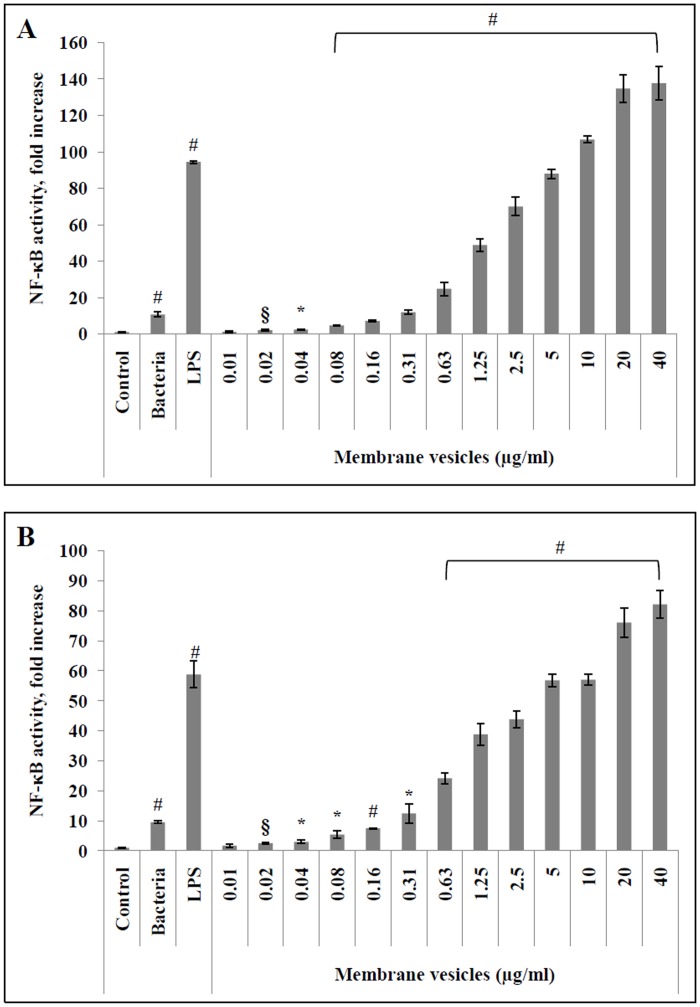
Quantification of NF-κB activation in U937-3xκB-LUC monocytes (A) and macrophage-like cells (B) by *S. suis* membrane vesicles (0.01 to 40 μg/ml), *E. coli* LPS (10 μg/ml), and S. suis P1/7 whole bacteria (MOI = 100). Results were considered significant at §: *p* < 0.05, *: *p* < 0.01, and #: *p* < 0.001 compared to unstimulated cells.

To explore the potential contribution of MVs to the inflammatory process associated with *S*. *suis* infections such as meningitis, NF-κB activition was monitored in monocytes and macrophages transfected with a luciferase reporter gene. NF-κB was dose-dependently activated by MVs following a 6-h stimulation of the monocytes and PMA-differentiated macrophages (Fig 5). More specifically, in the presence of 0.02 μg/ml (protein content) of MVs, NF-κB activity increased 2- and 2.5-fold compared to unstimulated monocytes and macrophage-like cells, respectively. In the presence of 40 μg/ml (protein content) MVs, NF-κB activity in monocytes and macrophages increased 137- and 82-fold, respectively, while *S*. *suis* P1/7 whole cells at an MOI of 100 increased NF-κB activity by approximately 10-fold in both monocytes and macrophage-like cells. Used as a positive control, *E*. *coli* LPS (10 μg/ml), which is a strong pro-inflammatory inducer, caused a 100- and 60-fold increase in NF-κB activity in monocytes and macrophages, respectively.

**Fig 6:**
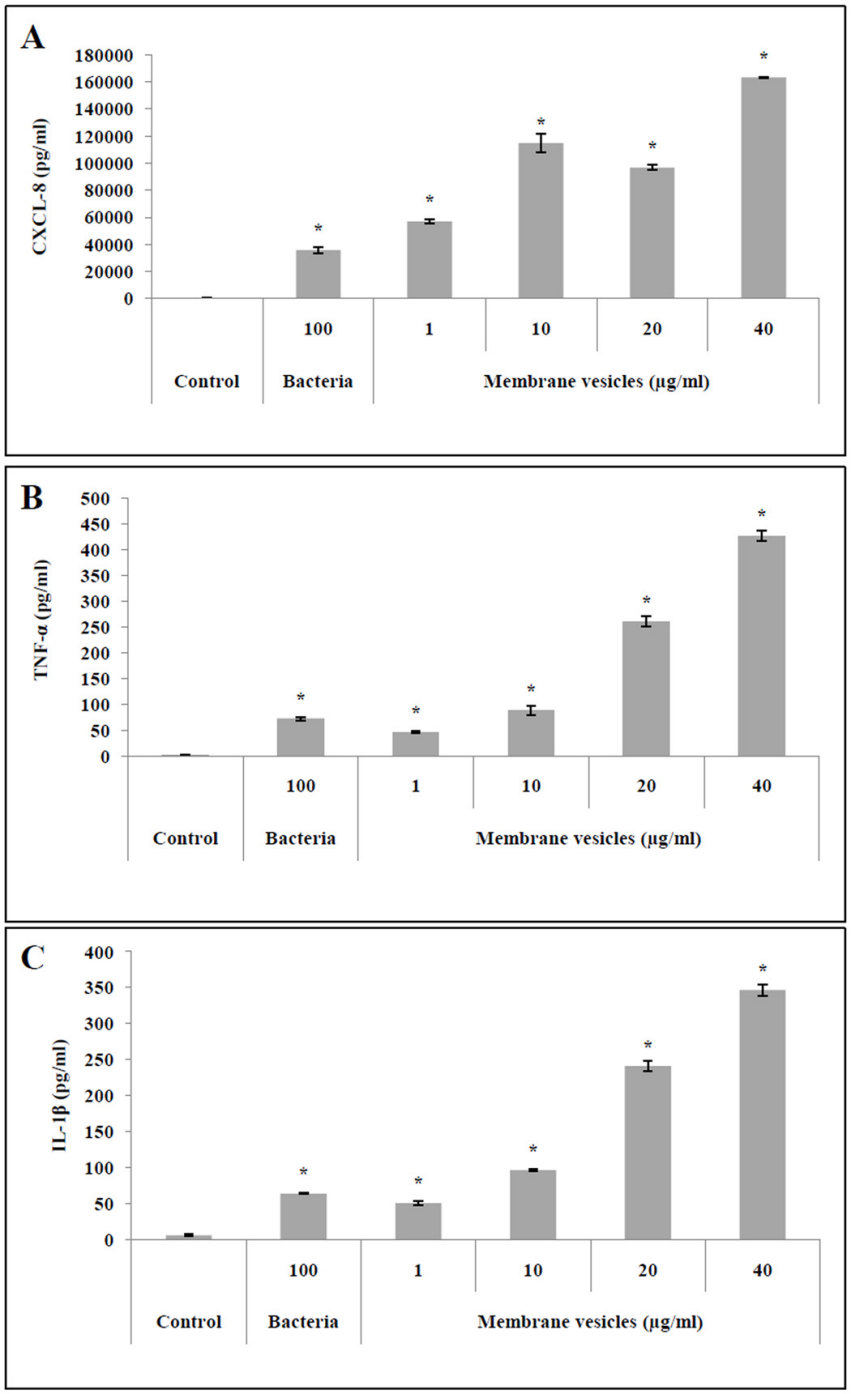
Quantification of CXCL-8 (A), TNF-α (B), and IL-1β (C) secretion by macrophage-like cells stimulated with *S. suis* membrane vesicles (1 to 40 μg/ml), *E. coli* LPS (10 μg/ml), and *S. suis* P1/7 whole bacteria (MOI = 100). Results were considered significant at *p < 0.05 compared to unstimulated cells.

PMA-differentiated U937 cells were stimulated in the presence of *S*. *suis* MVs in order to correlate the increased NF-κB activity observed in the U937-3xκB-LUC cell line with the secretion of pro-inflammatory cytokines. As shown in Fig 6, *S*. *suis* MVs dose-dependently increased the secretion of CXCL-8, TNF-α, and IL-1β. For example, 40 μg/ml protein content) of MVs increased the secretion of CXCL-8, TNF-α, and IL-1β by macrophage-like cells 4.6-, 5.9-, and 5.4-fold, respectively, compared to the amounts secreted when *S*. *suis* P1/7 whole cells (MOI = 100) alone were used to stimulate the macrophage-like cells.

## Discussion

Despite significant advances in our understanding of the pathogenic process of *S*. *suis* infections, major unresolved issues remain, and consequently additional studies are required to better characterize the pathogenic strategies of this swine and zoonotic pathogen. In the present study, we hypothesized that *S*. *suis* produces MVs as it has been observed with other pathogenic streptococci [20, 21]. Transmission electron microscopic observations of overnight cultures showed that *S*. *suis* P1/7 releases extracellular MVs during normal growth. MVs with diameters ranging from 13 to 130 nm were isolated from a culture supernatant of *S*. *suis* P1/7 using a simple differential centrifugation and filtration protocol. The MVs appeared to be coated by capsular material that may have bound to the vesicles during the blebbing process. Since MVs were isolated without the use of gradient centrifugation, one should not exclude that this capsular-like material could contain remaining cellular debris, even though isolated MVs were washed once in PBS. Given the relatively low vesicle yield, further studies are required to determine the growth conditions that will increase the production of MVs by *S*. *suis*.

A proteomic analysis of the *S*. *suis* MVs identified 46 proteins that participate in a broad array of functions, including molecule transport, protein, carbohydrate, and nucleotide metabolism, gene expression and protein folding processes. MVs produced by *S*. *aureus* [36] and *S*. *pneumoniae* [21] were reported to contain 143 and 211 proteins, respectively. Like *S*. *aureus* and *S*. *pneumoniae* MVs, *S*. *suis* MVs contain mainly cytoplasmic proteins as well as some membrane and cell wall proteins.

Nine proteins that are proven or suspected virulence factors were identified in *S*. *suis* MVs, including seven surface proteins (two membrane-bound: HtpsC [37], Ide [38]; and five cell wall-anchored: SspA [39, 40], SsnA [41, 42], Mrp [43], ApuA [44] and Eno [45]), and two extracellular proteins (Plr [46] and Sly [47, 48]). The two most abundant virulence factors in MVs were the subtilisin protease SspA and the cell wall-anchored DNase SsnA, which were also among the most abundant proteins overall in *S*. *suis* MVs. Biological assays confirmed the presence of both subtilisin and DNase activities in MVs. SspA can degrade the Aα chain of fibrinogen and has been suggested to play a key role in the pathogenicity of *S*. *suis* since an SspA-deficient mutant is avirulent in a standard mouse model [40]. Moreover, recombinant SspA induces the secretion of major pro-inflammatory cytokines and, when present in high concentrations, degrades CCL5 and IL-6 [49]. SsnA is the most important cell surface DNase in *S*. *suis* and may also be an important virulence factor since an SsnA-deficient mutant has been shown to be susceptible to predation in an amoebae virulence model [32]. Vesicle-associated DNase activity has been previously identified in OMVs secreted by the periodontopathogenic bacterium *Porphyromonas gingivalis* [50]. Given that SsnA allows bacteria to escape killing by NETs through the degradation of the DNA backbone [42], we investigated the ability of *S*. *suis* MVs to degrade NETs produced by PMA-stimulated neutrophils. *S*. *suis*-derived MVs dose-dependently degraded extracellular DNA, suggesting that MVs produced by *S*. *suis* may play an important role in the ability of the bacteria to escape NETs.

Given that extracellular vesicles can deliver bacterial effectors to host cells, several studies have shown that these structures can modulate the host immune response [51–53]. The monocytic U937-3xκB-LUC cell line was used to monitor the activation of the NF-κB signaling pathway in order to characterize the immunogenic properties of *S*. *suis* MVs. The MVs dose-dependently increased NF-κB activity in both monocytes and PMA-differentiated macrophage-like cells. This is in agreement with a study showing that OMVs produced by *Escherichia coli* can activate the NF-κB pathway in endothelial cells [51]. Since the NF-κB signaling pathway mediates the secretion of several pro-inflammatory cytokines [54], we assayed cytokine secretion by PMA-differentiated U937 cells stimulated with *S*. *suis* MVs. The MVs dose-dependently stimulated CXCL-8, TNF-α, and IL-1β secretion by the U937 cells, which is in agreement with the results reported by Jun et al. (2013), who showed that OMVs secreted by *Acinetobacter baumannii*, an opportunistic pathogen, elicit a pro-inflammatory response via surface-exposed proteins both *in vitro* and *in vivo* [55]. Gram-positive MVs not only carry virulence factors and bacterial effectors [15, 56], but can also act as delivery systems for these molecules towards host cells inducing pro-inflammatory pathways that can lead to cellular death [18, 36, 52]. The activation of host cells by *S*. *suis* MVs may result from the interactions of vesicular constituents, which likely contain pathogen-associated molecular patterns (PAMPs) with Toll-like receptors (TLRs) as previously shown for MVs secreted by *S*. *aureus* [52]. Since the host inflammatory response is very important in bacterial meningitis [57], the immuno-modulatory activity of *S*. *suis* MVs may contribute to the etiopathogenic process of the disease. The accumulation of pro-inflammatory mediators can affect the permeability of the blood brain barrier during meningitis. Increasing the permeability of the blood brain barrier facilitates the migration of bacteria to the central nervous system and may promote disease progression and severity [58].

To the best of our knowledge, while more than twenty-five Gram-negative bacterial species have been shown to produce OMVs [59], the release of MVs by Gram-positive bacteria has been reported for much fewer species, including *Bacillus anthracis* [15], *Bacillus subtilis* [16], *C*. *perfringens* [18], *L*. *monocytogenes* [17], *S*. *aureus* [12], *S*. *mutans* [20], and *S*. *pneumoniae* [21]. The present study reports for the first time that *S*. *suis* produces MVs that carry large amounts of proteins and active virulence factors and that may reach areas not accessible to whole bacteria. In addition, MVs may compete for antibodies and impede specific antibacterial immune defenses.

The emergence of drug-resistant strains of *S*. *suis* due to the widespread and inappropriate use of antibiotics by the swine industry is a major economic and public health concern [60]. Because of this, it is important to explore the fundamental pathogenic mechanisms of *S*. *suis* involved during infections in order to identify new candidates for treating *S*. *suis* infections and developing effective vaccines. Since *S*. *suis* MVs present a broad array of antigens, they may be a potential candidate for designing vaccines for passive antibody-based therapeutic strategies. Extracellular bacterial vesicles activate immune responses and induce immunological memories [11, 59]. For example, it has recently been shown that MVs released by *S*. *pneumoniae* induce a protective immune response and protect mice against infections by a virulent strain [21]. Further characterization of the composition and immunogenicity of *S*. *suis* MVs is required to better evaluate the potential of these structures for the development of an effective acellular vaccine to prevent *S*. *suis* infections.
